# Potential efficacy of therapies targeting intrahepatic lesions after sorafenib treatment of patients with hepatocellular carcinoma

**DOI:** 10.1186/s12885-016-2380-4

**Published:** 2016-05-31

**Authors:** Takeshi Terashima, Tatsuya Yamashita, Rika Horii, Kuniaki Arai, Kazunori Kawaguchi, Kazuya Kitamura, Taro Yamashita, Yoshio Sakai, Eishiro Mizukoshi, Masao Honda, Shuichi Kaneko

**Affiliations:** Faculty of Medicine, Institute of Medical, Pharmaceutical and Health Sciences, Kanazawa University, Kanazawa, Ishikawa 920-8641 Japan; Department of Gastroenterology, Kanazawa University Hospital, 13-1 Takara-machi, Kanazawa, Ishikawa 920-8641 Japan

**Keywords:** Hepatocellular carcinoma, Sorafenib, Subsequent therapy, Posttreatment survival, Hepatic arterial infusion chemotherapy

## Abstract

**Background:**

We investigated the contribution of subsequent therapy for advanced hepatocellular carcinoma refractory or intolerant to sorafenib. Further, we investigated the impact of sorafenib on overall survival using individual data.

**Methods:**

We reviewed the medical records of patients with advanced hepatocellular carcinoma treated with sorafenib. Survival after sorafenib treatment and overall survival were defined as the time when we discovered that patients were either refractory or intolerant to sorafenib and the period from the start of sorafenib treatment, respectively, until death during the study. We compared patients’ prognoses according to their subsequent treatment as follows: group A, therapies targeting intrahepatic lesions; group B, systemic therapies alone; group C, no subsequent therapy. We used linear regression analysis to determine whether there was an association with survival after sorafenib treatment and with overall survival.

**Results:**

Of 79 patients, 63 (79.7 %) received one or more subsequent therapies (44 and 19 patients in groups A and B, respectively). The five patients who survived more than two years after sorafenib treatment was discontinued responded to therapies targeting intrahepatic lesions. The median survival times of groups A, B, and C were 11.9 months, 5.8 months, and 3.6 months, respectively. Multivariate analysis revealed that group A, Child-Pugh score, serum α-fetoprotein level, and cause of failure of sorafenib treatment were independent prognostic factors for survival after sorafenib treatment. Individual survival after sorafenib treatment correlated highly with overall survival.

**Conclusions:**

Targeting intrahepatic lesions may be useful for treating patients with advanced hepatocellular carcinoma patients after sorafenib treatment is discontinued.

**Electronic supplementary material:**

The online version of this article (doi:10.1186/s12885-016-2380-4) contains supplementary material, which is available to authorized users.

## Background

Hepatocellular carcinoma (HCC) is the third leading cause of cancer-related mortality worldwide [1]. A variety of new imaging techniques detect HCC at early stages [2]. However, the number of patients with HCC who can be treated curatively is limited owing to impaired hepatic reserve and frequent metachronous recurrence to become difficult to treat. Therefore, the prognosis of patients with advanced HCC remains poor [3]. Sorafenib, an orally administered multikinase inhibitor that blocks tumor cell proliferation and angiogenesis, represents the only systemic drug that significantly improves overall survival (OS) of patients with advanced HCC [4]. Therefore, sorafenib is recognized as standard first-line therapy for advanced HCC [5, 6].

Previous analyses suggest that the survival of patients with HCC who are refractory or intolerant to sorafenib correlates highly with OS, whereas progression-free survival correlates less well with OS of patients with advanced HCC [7, 8]. These results are unexpected, because randomized trials fail to show that drugs that were administered after discontinuation of sorafenib treatment are effective for treating HCC [9]. Therefore, longer treatment with sorafenib may correlate directly with improvement of OS of patients with advanced HCC [10]. One of the possible reasons for the strong correlation between survival after sorafenib treatment and OS may be the beneficial role of subsequent therapy such as hepatic arterial infusion chemotherapy [11], transarterial chemoembolization [12], or continuation of sorafenib beyond progression [13] that increases survival after prolonged treatment with sorafenib. However, the effects of these treatments in clinical practice remain unclear.

The aim of the present study is to investigate treatment strategies and the contribution of subsequent therapies, particularly those targeting intrahepatic lesions, by identifying prognostic factors for patients with advanced HCC after sorafenib treatment in study 1. Moreover, we investigated the effect of posttreatment survival after sorafenib treatment on OS in study 2. We show here that this approach provides useful information for designing an optimal treatment strategy for advanced HCC after treatment with sorafenib.

## Methods

### Patients

The subjects were consecutive patients with advanced HCC treated with sorafenib monotherapy at the Kanazawa University Hospital between June 2009 and October 2014. All patients underwent dynamic computed tomography (CT) to diagnose and assess the extent of the cancer. HCC was diagnosed according to the guidelines of the American Association for the Study of Liver Disease guidelines [14]. Patients received 400 mg of sorafenib orally twice daily. Treatment was temporarily interrupted or reduced according to toxicity and was continued until the confirmation of tumor progression or the occurrence of unacceptable adverse effects. Patients were considered as candidates for subsequent therapy in study 1 if, at the time of their refractory response or intolerance to sorafenib, their characteristics were as follows: Eastern Cooperative Oncology Group performance status = 0 or 1; appropriate function of major organs, including bone marrow, kidney, and cardiac function; and hepatic reserve with Child-Pugh class A or B. Of the patients included in study1, those who were alive at the last visit before the time of analysis were excluded, and only patients with confirmed survival data were included in study 2.

### Evaluation

To assess the antitumor effects of sorafenib, dynamic CT was conducted every six weeks during treatment and at the time of a refractory response or intolerance to sorafenib. Responses were assessed according to the Response Evaluation Criteria in Solid Tumors ver. 1.1 [15]. We reviewed patients’ medical records and collected demographic, clinical, and laboratory data including age, sex, Eastern Cooperative Oncology Group performance status, history of viral infection, factors related to hepatic reserve associated with Child-Pugh classification, imaging data (vascular invasion and extrahepatic spread of HCC), and tumor markers at the time of a refractory response or intolerance to sorafenib. We further investigated the cause of failure of sorafenib treatment (refractory response or intolerance).

“Survival after sorafenib”, time to treatment failure of sorafenib treatment, and OS was defined as follow in this study. Survival after sorafenib treatment was defined as the time of a refractory response or intolerance to sorafenib until death [16]. “Time to treatment failure of sorafenib treatment” was defined as the time from the start of sorafenib treatment until the time of a refractory response or intolerance to sorafenib. “OS” was defined as the period from start of sorafenib treatment until death and that is sum of time to treatment failure of sorafenib treatment and survival after sorafenib treatment.

### Statistical analysis

We first divided the patients into three groups according to their treatment after sorafenib treatment as follows. Those who received one or more therapies targeting intrahepatic lesions, including hepatic arterial infusion chemotherapy, transarterial chemoembolization, radio frequency ablation, and radiotherapy for intrahepatic lesion were classified as group A. Those who received only systemic therapy, including systemic cytotoxic chemotherapy, molecular targeted therapy, immunotherapy, and radiotherapy for extrahepatic lesions were classified as group B. Those who did not receive any subsequent therapy were classified as group C.

Categorical variables were compared using the chi-squared test when appropriate. For univariate analysis, the cumulative survival frequencies were calculated using Kaplan–Meier analysis that considered different clinical factors likely associated with survival after sorafenib treatment, and the differences were evaluated using the log-rank test. Only variables with *p* < 0.1 in univariate analysis were subsequently evaluated in multivariate analysis using the Cox’s proportional hazards regression model, and *p* < 0.05 was considered statistically significant. The relationship between OS and either survival after sorafenib treatment or time to treatment failure of sorafenib treatment were estimated using weighted linear regression analysis. All statistical analyses were performed using the SPSS statistical software program package (SPSS Inc., Chicago, IL, USA).

This was non-invasive retrospective observation study, and then, the need for individual consent is deemed unnecessary according to Japanese nation regulation, Ethical Guidelines for Medical and Health Research Involving Human Subjects (available: http://www.mhlw.go.jp/file/06-Seisakujouhou-10600000-Daijinkanboukouseikagakuka/0000080278.pdf). The protocol of this study was approved by the institutional review board at Kanazawa University and the study was conducted in accordance with the Declaration of Helsinki.

## Results

### Patient characteristics

**Fig. 1:**
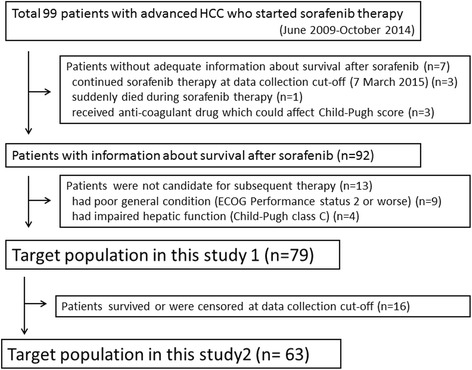
Study population. The target population of this study comprised patients with advanced HCC pretreated with sorafenib and those who were considered as candidates for subsequent therapy and were included in study 1. Patients who died before the analysis were included in study 2

The data collection ended March 7, 2015. We reviewed retrospectively 99 consecutive patients with advanced HCC who were treated with sorafenib monotherapy in our institution between June 2009 and October 2014. We were unable to acquire sufficient information about survival after sorafenib treatment for seven patients, and 13 patients did not meet the selection criteria for subsequent therapy. Thus, 79 subjects were included in study 1 (Fig. 1).

The median time to treatment failure of sorafenib treatment of these 79 patients was 2.56 months (range, 0.03–17.75 months) (Additional file 1: Figure S1). The response rate and tumor control rate for sorafenib treatment were 2.5 % and 53.2 %, respectively, in accordance with Response Evaluation Criteria in Solid Tumors ver1.1. Sorafenib was terminated because of tumor progression (refractory to sorafenib) for 59 patients (74.7 %) and unacceptable adverse effects (intolerant to sorafenib) for 20 patients (25.3 %).

### Treatment strategy after discontinuation of sorafenib treatment

The median follow-up period for survival after sorafenib treatment and OS were 6.6 months and 9.8 months, respectively. Sixty-three patients (79.7 %) received one or more subsequent therapies after sorafenib treatment, including hepatic arterial infusion chemotherapy (*n* = 37), systemic cytotoxic chemotherapy (*n* = 22), transarterial chemoembolization (*n* = 12), molecular targeted therapy or continuous administration of sorafenib (*n* = 11), immunotherapy (*n* = 10), radiofrequency ablation (*n* = 7), and radiotherapy of intrahepatic lesions (*n* = 4). The remaining 16 patients (20.3 %) received appropriate information about available treatment options, which they declined. There were 44, 19, and 16 patients were classified as group A (those who received one or more therapies targeting intrahepatic lesions), B (those who received only systemic therapy), and C (those who did not receive any subsequent therapy), respectively.

Patients’ demographics at the time of a refractory response or intolerance to sorafenib are summarized in Table 1. There was no statistically significant difference among groups.

**Table 1:** Patient characteristics at the time of a refractory response or intolerance to sorafenib treatment

	Total	group A	group B	group C	*P* value^*^
	(*n* = 79)	(*n* = 44)	(*n* = 19)	(*n* = 16)	
Age, years					0.67
≥68	43 (54.4)	23 (52.3)	12 (63.2)	8 (50.0)	
Gender					0.72
Male	71 (89.9)	39 (88.6)	18 (94.7)	14 (87.5)	
ECOG performance status					0.46
0	50 (63.3)	29 (65.9)	13 (68.4)	8 (50.0)	
1	29 (36.7)	15 (34.1)	6 (31.6)	8 (50.0)	
hepatitis B surface antigen					0.33
Positive	27 (34.2)	16 (36.4)	4 (21.1)	7 (43.8)	
hepatitis C virus antibody					0.82
Positive	34 (43.0)	20 (45.5)	7 (36.8)	7 (43.8)	
Child-Pugh class (Child-Pugh score)					0.064
A (5)	21 (26.6)	11 (25.0)	7 (36.8)	3 (18.8)	
A (6)	27 (34.2)	19 (43.2)	6 (31.6)	2 (12.5)	
B	31 (39.2)	14 (31.8)	6 (31.6)	11 (68.8)	
Vascular invasion					0.097
Positive	26 (32.9)	15 (34.1)	3 (15.8)	8 (50.0)	
Extra-hepatic spread					0.62
Positive	43 (54.4)	22 (50.0)	12 (63.2)	9 (56.3)	
AFP ^a^, n (%)					0.87
≥400 ng/mL	29 (36.7)	17 (38.6)	6 (31.6)	6 (37.5)	
Cause of failure of sorafenib treamtnet					0.080
Tumor progression	60 (75.9)	37 (84.1)	14 (73.7)	9 (56.3)	
Adverse effect	19 (24.1)	7 (15.9)	5 (26.3)	7 (43.8)	
Time to treatment failure of sorafenib, months					0.059
<2.56	39 (49.4)	20 (45.5)	7 (36.8)	12 (75.0)	
≥2.56	40 (50.6)	24 (54.5)	12 (63.2)	4 (25.0)	

^a^ AFP α-fetoprotein

*chi-squared test

### Survival after sorafenib according to subsequent therapy

**Fig. 2:**
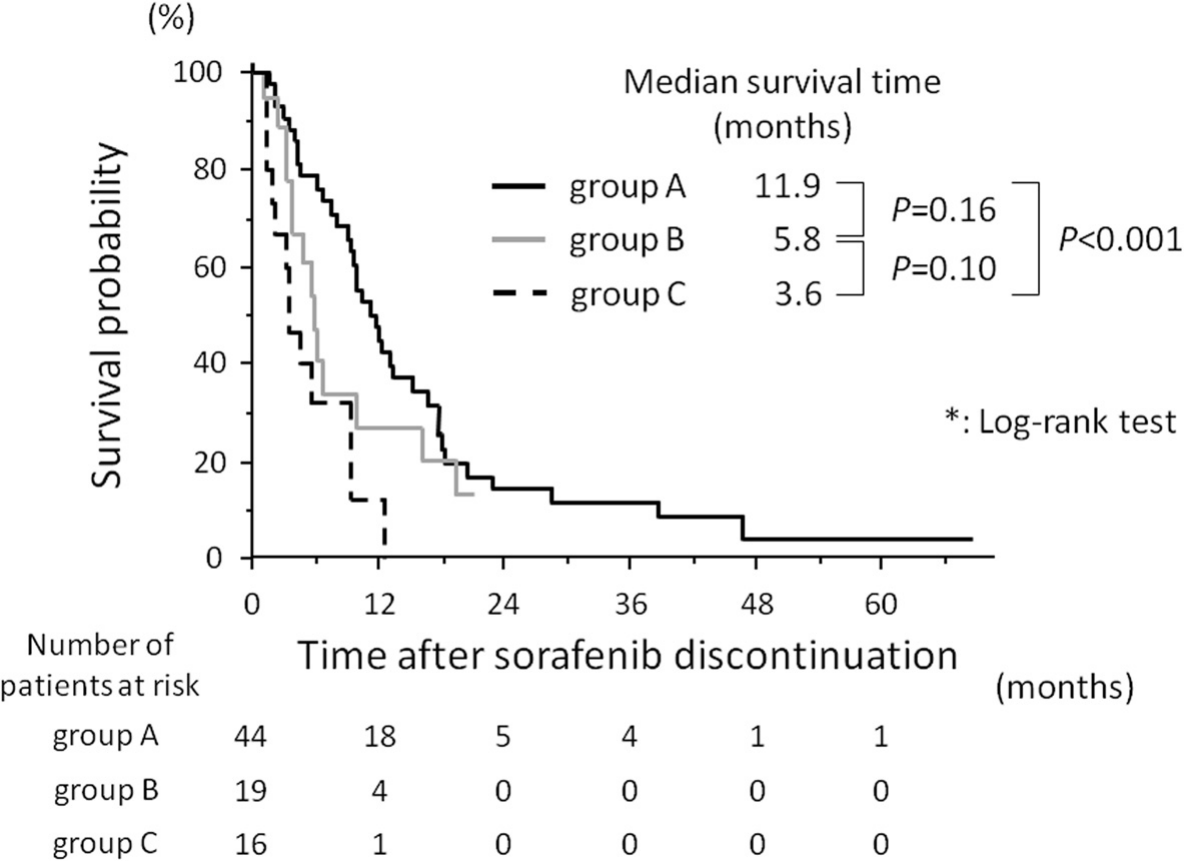
Kaplan–Meier analysis of survival. The median survival after sorafenib treatment of patients in group A (patients receiving therapies targeting intrahepatic lesions; black line), group B (patients receiving systemic therapy alone, gray line), and group C (no subsequent therapy, dashedline) were 11.9, 5.8 and 3.6 months, respectively. Survival after sorafenib treatment of group A was significantly longer compared with that of group C (*p* < 0.001)

Median survival times after a refractory response or intolerance to sorafenib for patients in groups A, B, and C were 11.9, 5.8, and 3.6 months, respectively. Survival after sorafenib treatment of group A was significantly longer compared with that of group C (*p* < 0.001) (Fig. 2).

Taking into consideration the median follow-up period as described above, we consider the patients who survived longer than two years after discontinuation of sorafenib treatment as long survivor, and their characteristics are shown in Table 2. They all responded to subsequent therapies targeting intrahepatic lesions, and three of the four patients who received hepatic arterial infusion chemotherapy achieved a partial response.

**Table 2:** Clinical characteristics of patients who survived longer than two years after sorafenib treatment

Child-Pugh class (score)	AFP ^a^ (ng/mL)	Cause of failure of sorafenib treatment	Subsequent therapy			Survival after sorafenib treatment (months), Status at analysis
			Therapies targeting intra-hepatic lesion	Response^b^ to hepatic arterial infusion chemotehrapy	Systemic therapy	
A (6)	15	adverse effect	hepatic arterial infusion chemotherapy, radio frequent ablation	Partial response	Radiotherapy (for bone metastases), cytotoxic chemotherapy, immunotherapy	28.4 dead
A (5)	16	adverse effect	hepatic arterial infusion chemotherapy	Partial response	cytotoxic chemotherapy, sorafenib re-administration	68.5 alive
A (5)	17688	tumor progression	radio frequent ablation	-	Radiotherpay (for brain metastases), immunotherapy	46.8 dead
B (9)	11	adverse effect	hepatic arterial infusion chemotherapy, transarterial chemoembolization, radio frequent ablation	Partial response	cytotoxic chemotherapy	38.6 dead
A (5)	964	tumor progression	hepatic arterial infusion chemotherapy, transarterial chemoembolization radio frequent ablation, radiotherapy	Stable disease	-	40.0 alive

^a^ AFP α-fetoprotein

^b^ Response Evaluation Criteria in Solid Tumors ver1.1

### Prognostic factors of survival after sorafenib treatment

Univariate analyses identified four of the 11 variables that were significantly associated with prognostic factors of survival after sorafenib treatment (Table 3) as follows: Child-Pugh class, serum AFP level, cause of failure of sorafenib treatment as well as subsequent therapies targeting intrahepatic lesions. Independent factors that were unfavorable for survival after sorafenib treatment were as follows: no subsequent therapy, subsequent systemic therapies alone, Child-Pugh class B, Child-Pugh score 6, 400 ng/mL or higher serum AFP level, and discontinuation of sorafenib due to tumor progression (Table 3).

**Table 3:** Prognostic factors affecting survival after sorafenib treatment

	n	Median survival after sorafenib, months	Univariate *P* value*	Hazard ration (95% confidence interval)	Multivariate *P* value**
Age, years					
≥68	43	9.7	0.65		
<68	36	9.3			
Gender					
Male	71	9.3	0.78		
Female	8	9.5			
hepatitis B surface antigen					
Positive	27	10.5	0.67		
Negative	52	9.4			
hepatitis C virus antibody					
Negative	45	9.2	0.90		
Positive	34	9.5			
Child-Pugh class (Child-Pugh score)					
B	31	4.7	0.070	2.999 (1.478-6.087)	0.009
A (6)	27	11.9		2.328 (1.109-4.884)	0.025
A (5)	21	9.9			
Vascular invasion					
Positive	26	6.7	0.29		
Negative	53	9.9			
Extra-hepatic lesion					
Positive	43	6.6	0.12		
Negative	36	9.7			
AFP ^a^, ng/mL					
≥400	29	4.3	0.032	2.207 (1.230-3.958)	0.008
<400	50	9.9			
Cause of failure of sorafenib treatment					
Tumor progression	60	8.1	0.045	2.331 (1.168-4.650)	0.016
Adverse effect	19	13.5			
Time to treatment failure of sorafenib treatment, months					
<2.56	39	8.1	0.49		
≥2.56	40	9.9			
Subsequent therapy					
group C	16	3.6	0.001	5.805 (2.684-12.553)	<0.001
group B	19	5.8		2.628 (1.309-5.278)	0.007
group A	44	11.9			

^a^ AFP: α-fetoprotein

*Log-rank test, **Cox’s proportional hazards regression model

### Correlation between OS and survival after sorafenib treatment or time to treatment failure

**Fig. 3:**
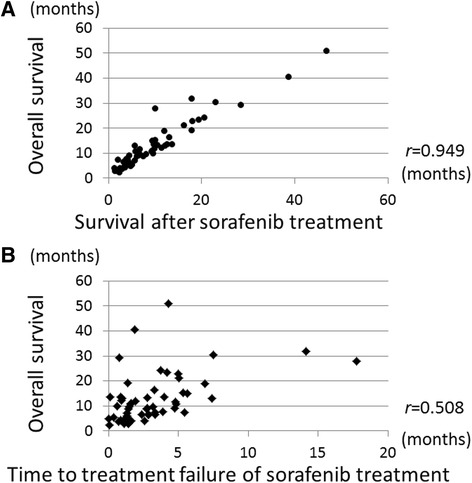
Linear regression analysis of overall survival and survival after sorafenib treatment and time to treatment failure of sorafenib treatment. **a** Survival after sorafenib treatment correlated significantly with overall survival (*r* = 0.949). **b** Time to treatment failure of sorafenib treatment correlated with overall survival (*r* = 0.508) as well

Sixteen patients were alive at the last visit before the time of the analysis, and the correlation between OS and survival after sorafenib treatment or time to treatment failure of sorafenib treatment was assessed among the remaining 63 patients with confirmed data (Fig. 1). Median OS and survival after sorafenib treatment were 10.1 months and 6.7 months. The OS data were plotted against survival after sorafenib treatment (Fig. 3a) and time to treatment failure of sorafenib treatment (Fig. 3b). We found that survival after sorafenib treatment highly correlated with OS (*r* = 0.949), while time to treatment failure of sorafenib treatment did not well correlate with OS (*r* = 0.508). These differences were more apparent in the subgroup of Child-Pugh class A compared with B, BCLC stage B compared with C, discontinuation of sorafenib because of adverse effects compared with tumor progression, and any subsequent therapy compared with no subsequent therapy (Table 4).

**Table 4:** Analysis of overall survival and survival after sorafenib treatment or time to treatment failure of sorafenib treatment

	n	OS ^a^ and survival after sorafenib treatment		OS ^a^ and time to treatment failure of sorafenib treatment	
		*R*	*P* value*	*r*	*P* value*
All patients					
	63	0.949	<0.001	0.508	<0.001
Child-Pugh class (Child-Pugh score)					
A (5)	14	0.992	<0.001	0.176	0.56
A (6)	21	0.944	<0.001	0.687	<0.001
B	28	0.920	<0.001	0.570	0.001
BCLC stage					
B	16	0.968	<0.001	0.313	0.24
C	47	0.940	<0.001	0.555	<0.001
AFP ^b^, ng/mL					
≥400	25	0.947	<0.001	0.490	0.012
<400	38	0.948	<0.001	0.500	0.001
Cause of failure of sorafenib treatment					
Tumor progression	49	0.939	<0.001	0.596	<0.001
Adverse effect	14	0.989	<0.001	0.363	0.21
Subsequent therapy					
No	13	0.880	<0.001	0.428	0.15
Any	50	0.947	<0.001	0.471	<0.001

^a^ OS overall survival

^b^ AFP α-fetoprotein

*linear regression analysis

## Discussion

We show here that therapies targeting intrahepatic lesions administered after discontinuing sorafenib treatment represented one of the independent prognostic factors for patients with advanced HCC. This finding has important implications for designing treatment strategies for such patients. Factors such as general health, hepatic reserve, and tumor-associated factors such as vascular invasion and serum AFP levels predict the outcomes of patients with advanced HCC [17], and the progression pattern and cause of failure of sorafenib treatment stratifies patients’ outcomes [18]. Our present results show that subsequent therapy targeting intrahepatic lesions was a prognostic factor independent of Child-Pugh score, serum AFP level, and the reason for discontinuing sorafenib treatment. A report refers to the possible role of subsequent therapy [17]; however, they do not consider patients’ conditions or other confounding factors. We excluded patients whose performance status was poor, and their liver function was decompensated. Therefore, the objectives were restricted to patients considered as candidates for any subsequent therapy in this study.

Our findings suggest that treatment procedures targeting intrahepatic lesions were useful for increasing survival after sorafenib treatment. Although extrahepatic lesions are often observed in patients with advanced HCC, they are only 7.6 % of cause of death [19]. Moreover, the presence of intrahepatic lesions is one of the prognostic factors for radiologic progression and OS for patients with HCC, even those with extrahepatic spread treated with sorafenib [13]. Therefore, good control of intrahepatic lesions may affect the prognosis of these patients [20].

Although local therapies targeting intrahepatic lesions such as hepatic arterial infusion chemotherapy, transarterial chemoembolization, radiofrequency ablation, or radiotherapy are conventionally administered to reduce the tumor burden of patients after sorafenib treatment, no prospective trials, to our knowledge, verify their benefit for survival. Our present results indicate that such therapies may serve as promising treatment strategies, even after patients are administered systemic sorafenib.

The results of the present nonrandomized study should be interpreted with caution because of potential confounding factors affecting treatment strategy. Some different factors were observed between groups including the hepatic reservation, cause of sorafenib discontinuation, and time to treatment failure of sorafenib treatment, although the differences were not statistically significant (Table 1). Further, the outcomes were possibly affected by unidentified factors that were unique to group C. However, although the retrospective design did not allow us to reach a definitive conclusion, the results of multivariate analysis support a beneficial effect of therapies that target intrahepatic lesions, and the significance of the effects of these treatments should be noticeable under certain circumstances. For example, the control group should be treated with best available therapy that target intrahepatic lesions in clinical trials designed to evaluate new agents. The proportion of patients receiving therapies that target intrahepatic lesions subsequent to sorafenib treatment should be considered in the analysis of the outcomes of clinical trials. More aggressive application of subsequent therapy that target intrahepatic lesions should be considered to target intrahepatic lesions in clinical practice.

The participants of a workshop held at the 50th Annual Meeting of the Liver Cancer Study Group of Japan issued a list of five factors related to long-term survival of patients with HCC that includes effective post-sorafenib options [21]. The long-term effect of sorafenib is restricted owing to its static antitumor activity and numerous types of toxicities [4, 22], which are consistent with our finding that the sorafenib treatment was successful for 1 year for only two patients (2.5 %) (Additional file 1: Figure S1). In contrast, others reported responses of approximately 30 % of patients treated with hepatic arterial infusion chemotherapy, and long-term survival was expected in such patients [11]. It is important to note that hepatic arterial infusion chemotherapy is effective when administered after sorafenib and its efficacy is independent of sorafenib treatment.

Finally, we believe that our present finding that survival after sorafenib treatment correlated highly with OS, which was determined according to individual data, is very important. Further, survival after sorafenib treatment was not significantly stratified according to the efficacy of sorafenib treatment. These findings suggest that patients with HCC who did not obtain a satisfactory benefit from sorafenib treatment may benefit from subsequent therapies. The significance of survival after sorafenib treatment should be noted for the conditions as follows: The duration of survival after sorafenib treatment should be carefully estimated when calculating the sample size of clinical trials, and survival after sorafenib treatment should be considered in the analysis of the outcomes of clinical trials.

Our study has several limitations such as its retrospective design and the acquisition of data from a single institution. A properly designed prospective trial with a large number of subjects is required to confirm the significance of the effects of subsequent therapies targeting intrahepatic lesions.

## Conclusion

In conclusion, survival after sorafenib treatment was dependent on subsequent therapies targeting intrahepatic lesions, Child-Pugh score, serum AFP level, and the reason for discontinuing sorafenib treatment. Conventional post-sorafenib therapies, particularly those targeting intrahepatic lesions, such as hepatic arterial infusion chemotherapy, palliative transarterial chemoembolization, radiofrequency ablation, or radiotherapy may be useful. Therefore, we recommended them for patients with advanced HCC after sorafenib treatment is discontinued.

## Abbreviations

AFP, α-fetoprotein; CT, computed tomography; HCC, hepatocellular carcinoma; OS, overall survival.

## Additional file

Additional file 1: Figure S1. Kaplan–Meier analysis of time to treatment failure of sorafenib.treatment. Median time to treatment failure for all patients was 2.56 months. (TIF 32 kb)
