# Supplementary figures and images for: Potential efficacy of therapies targeting intrahepatic lesions after sorafenib treatment of patients with hepatocellular carcinoma
Source: BMC Cancer. 2016 May 31;16:338. doi: 10.1186/s12885-016-2380-4 (PMC4886418; doi:10.1186/s12885-016-2380-4)

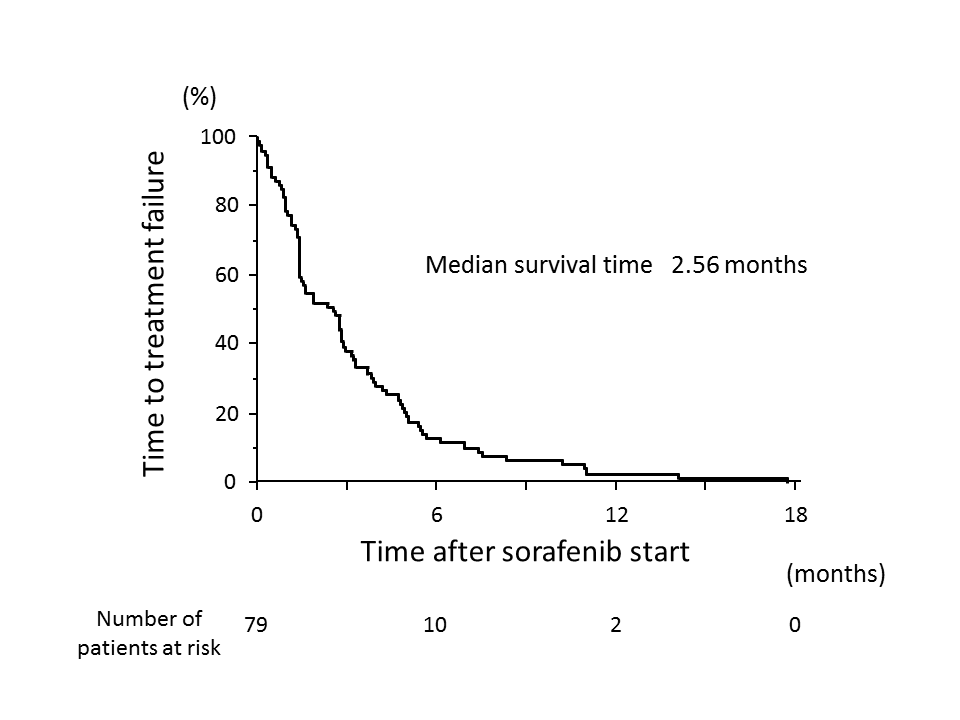

Supplement: Additional file 1: Figure S1. — Kaplan–Meier analysis of time to treatment failure of sorafenib.treatment. Median time to treatment failure for all patients was 2.56 months. (TIF 32 kb) [file 12885_2016_2380_MOESM1_ESM.tif]
